# Observational Assessment of Changes in Earth’s Energy Imbalance Since 2000

**DOI:** 10.1007/s10712-024-09838-8

**Published:** 2024-05-07

**Authors:** Norman G. Loeb, Seung-Hee Ham, Richard P. Allan, Tyler J. Thorsen, Benoit Meyssignac, Seiji Kato, Gregory C. Johnson, John M. Lyman

**Affiliations:** 1 NASA Langley Research Center, Hampton, VA 23681-2199 USA; 2 Analytical Mechanics Associates (AMA), Hampton, VA 23666 USA; 3 Department of Meteorology and National Centre for Earth Observation, University of Reading, Reading, RG6 6ET UK; 4 LEGOS, Université de Toulouse, CNES, CNRS, UPS, IRD, Toulouse, 31400 France; 5 NOAA/Pacific Marine Environmental Laboratory, Seattle, WA 98115 USA; 6 CIMAR, University of Hawaii, Honolulu, HI 96822 USA

**Keywords:** Earth’s energy imbalance, Climate change, Clouds, Satellite, Earth radiation budget

## Abstract

Satellite observations from the Clouds and the Earth’s Radiant Energy System show that Earth’s energy imbalance has doubled from 0.5 ± 0.2 Wm^−2^ during the first 10 years of this century to 1.0 ± 0.2 Wm^−^^2^ during the past decade. The increase is the result of a 0.9 ± 0.3 Wm^−2^ increase absorbed solar radiation (ASR) that is partially offset by a 0.4 ± 0.25 Wm^−2^ increase in outgoing longwave radiation (OLR). Despite marked differences in ASR and OLR trends during the hiatus (2000–2010), transition-to-El Niño (2010–2016) and post-El Niño (2016–2022) periods, trends in net top-of-atmosphere flux (NET) remain within 0.1 Wm^−2^ per decade of one another, implying a steady acceleration of climate warming. Northern and southern hemisphere trends in NET are consistent to 0.06 ± 0.31 Wm^−2^ per decade due to a compensation between weak ASR and OLR hemispheric trend differences of opposite sign. We find that large decreases in stratocumulus and middle clouds over the sub-tropics and decreases in low and middle clouds at mid-latitudes are the primary reasons for increasing ASR trends in the northern hemisphere (NH). These changes are especially large over the eastern and northern Pacific Ocean, and coincide with large increases in sea-surface temperature (SST). The decrease in cloud fraction and higher SSTs over the NH sub-tropics lead to a significant increase in OLR from cloud-free regions, which partially compensate for the NH ASR increase. Decreases in middle cloud reflection and a weaker reduction in low-cloud reflection account for the increase in ASR in the southern hemisphere, while OLR changes are weak. Changes in cloud cover in response to SST increases imply a feedback to climate change yet a contribution from radiative forcing or internal variability cannot be ruled out.

**Article Highlights**

- Satellite observations reveal that global mean net flux (NET) at the top-of-atmosphere (or equivalently, Earth’s energy imbalance) has doubled during the first twenty years of this century. The increase is associated with a marked increase in absorbed solar radiation (ASR) that is partially offset by an increase in outgoing longwave radiation (OLR)
- While ASR and OLR changes within sub-periods corresponding to the hiatus (03/2000–05/2010), transition-to-El Niño (06/2010–05/2016), and post-El Niño (06/2016–12/2022) vary substantially, NET flux changes are remarkably stable (within 0.1 Wm^−2^ per decade), implying a steady acceleration of climate warming
- The increase in ASR is associated with decreases in stratocumulus and middle cloud fraction and reflection in the Northern Hemisphere, and decreases in middle cloud reflection in the Southern Hemisphere. The cloud changes are especially large in areas with marked increases in sea-surface temperature, such as over the eastern and northern Pacific Ocean
- Continued monitoring of Earth’s radiation budget and new and updated climate model simulations are critically needed to understand how and why Earth’s climate is changing at such an accelerated pace

## Introduction

Earth’s radiation budget (ERB) describes how radiant energy is exchanged between Earth and space and how it is distributed within the climate system. The balance between incoming solar radiant energy absorbed by Earth and outgoing thermal infrared radiation emitted to space (also called Earth’s Energy Imbalance, or EEI) determines whether Earth heats up or cools down (Hansen et al. 2005; Trenberth et al. 2014). A positive EEI is concerning as the extra energy added to the climate system leads to warming of the oceans, land and atmosphere, sea level rise, melting of snow and ice, and shifts in atmospheric and oceanic circulations (von Schuckmann et al. 2016). Approximately 89% of this additional heat is stored in the ocean, while the rest warms the land (5%) and atmosphere (2%) and melts ice (4%) (von Schuckmann et al. 2023).

Multiple lines of evidence show that EEI is increasing. These include an in situ based Earth heat inventory that quantifies how much heat has accumulated in the Earth system and where the heat is stored (von Schuckmann et al. 2023; Minière et al. 2023; Li et al. 2023; Storto and Yang 2024; Cheng et al. 2024), satellite observations of top-of-atmosphere (TOA) radiative fluxes from the Clouds and the Earth’s Radiant Energy System (CERES) (Loeb et al. 2021a), and satellite measurements of sea level and ocean mass change (Hakuba et al. 2021; Meyssignac et al. 2023; Marti et al. 2023). In situ based Earth heat inventory observations of global ocean heat content (OHC) and non-ocean components (atmosphere, land and cryosphere) indicate a robust acceleration of Earth system heating since 1960 (von Schuckmann et al. 2023; Minière et al. 2023; Li et al. 2023; Storto and Yang 2024; Cheng et al. 2024). The acceleration rate for 1960–2020 is 0.15 ± 0.05 Wm^−2^ dec^−1^ and 0.30 ± 0.28 Wm^−2^ dec^−1^ for the more recent period between 2002 and 2020 (Minière et al. 2023). The latter is consistent within uncertainty with satellite observations of TOA net flux (Loeb et al. 2021a, 2022). In a comparison of CERES EEI with 18 OHC products derived from in situ, geodetic satellite observations, and ocean reanalyses for 2005–2019, Hakuba et al. (2024, this collection) show that while there is much spread in ocean heat uptake (OHU) and the rate of increase in OHU among the different analyses, the main reason for this spread is inadequate spatial–temporal sampling of the ocean. Datasets with better ocean coverage by filling in data sparse regions with satellite data or physical models (reanalyses) more closely match TOA net flux variability from CERES and show a positive trend in OHU that is similar in magnitude to CERES. It’s worth noting that better sampling does not always guarantee better results. Loeb et al. (2022) argue that in the case of ocean reanalyses, achieving reliable temporal fidelity also depends upon model bias and whether new data are introduced/removed from the time series.

Few studies have examined what is driving the EEI increase since 2000. Raghuraman et al. (2021) used Coupled Model Intercomparison Project Phase 6 (CMIP6) (Eyring et al. 2016) simulations from the Geophysical Fluid Dynamics Laboratory Coupled/Atmospheric Model 4.0 (GFDL CM4/AM4) (Zhao et al. 2018; Held et al. 2019) to assess the contributions of internal variability, effective radiative forcing (ERF) and climate feedbacks on the CERES trend. They conclude that the positive EEI trend can only be explained if the simulations account for the increase in anthropogenic radiative forcing and associated climate response since 2000. This is confirmed with four additional CMIP6 models by Hodnebrog et al. (2024), who further showed that effective radiative forcing due to anthropogenic aerosol emission reductions contributes 0.2 ± 0.1 Wm^−2^ dec^−1^ to the trend in EEI. Kramer et al. (2021) used satellite data to infer instantaneous radiative forcing, providing observational evidence that radiative forcing is a major factor behind the EEI trend. Unfortunately, the number of assessments of the observed EEI trend are limited because the CMIP6 protocol ends in 2014. Schmidt et al. (2023) propose a new atmosphere only model intercomparison, CERESMIP, that targets the CERES period using updated sea-surface temperatures (SSTs), forcings and emissions through 2021. These new atmospheric model intercomparison project (AMIP) simulations will greatly expand the number of models available for model–observation comparisons and attribution studies of the EEI trend.

An observation-based partial radiative perturbation (PRP) analysis based upon the methodology of Thorsen et al. (2018) indicates that the CERES trend in EEI since 2000 is manifested in the data through changes in cloud, water vapor, trace gases, surface albedo and aerosols, which combine to increase TOA net downward radiation in excess of a negative contribution from increasing temperature (Loeb et al. 2021a). These changes are a consequence of the combined effects of climate forcing, feedback, and internal variability. To date, there has not been a thorough analysis of how different cloud types contribute to the observed changes in EEI. Loeb et al. (2021a) show that there is a large contribution by clouds to absorbed solar radiation changes and a weaker contribution to outgoing longwave radiation changes of opposite sign, but it does not attribute these to any particular cloud type. Furthermore, Loeb et al. (2021a) note substantial variations in TOA radiation during different sub-periods within the CERES record associated with internal variability.

In the following, we provide an observational assessment of TOA radiation changes that updates prior analyses by considering the period from 2000 to 2022 using CERES data products (Sect. 3.1). We examine the global, zonal and regional variations and trends in TOA radiation both for the entire CERES period and sub-periods corresponding to the hiatus (2000–2010), transition-to-El Nino (2010–2016), and post-El Nino (2016–2022) to highlight TOA radiation changes across periods of markedly different internal variability (Sect. 3.2). We also use the new CERES FluxByCldTyp (FBCT) data product (Sun et al., 2022) to quantify the contribution to TOA radiation changes by different cloud types using a cloud classification scheme based upon cloud types provided in FBCT (Sect. 3.3). Finally, we discuss some of the challenges associated with isolating the underlying processes that contribute to changes in TOA radiation from observations alone (Sect. 4).

## Data and Methods

### TOA Radiation and Cloud Datasets

Anomalies in TOA radiation components relative to their seasonal cycles are determined from the CERES Energy Balanced and Filled (EBAF) Ed4.2 product (Loeb et al. 2018) for 03/2000–12/2022. The anomalies are determined by differencing the average in a given month from the average of all years of the same month. Throughout the paper, anomalies are defined positive downwards (hence the naming convention “–OLR” to indicate that an increase in OLR corresponds to a loss of energy relative to climatology). Trends are determined from monthly anomalies using least squares linear regression and uncertainties in the trends follow the approach described in Loeb et al. (2022). The EBAF product uses an objective constrainment algorithm (Loeb et al. 2009) to adjust shortwave (SW) and longwave (LW) TOA radiative fluxes within their ranges of uncertainty to anchor global net TOA flux to an in situ estimate of the global mean EEI from mid-2005 to mid-2015 (Johnson et al., 2016). Use of this approach to anchor the satellite-derived EEI does not impact the variability and trends in the data (Loeb et al. 2018). The EBAF product provides two clear-sky fluxes, one for cloud-free portions of a region and a second for the total region. The latter was introduced to provide an observation-based clear-sky flux defined in the same way as climate models (Loeb et al. 2020). Here we only consider clear-sky fluxes for cloud-free areas of a region and use that to compute cloud radiative effect (CRE), defined as the difference between all-sky and clear-sky downward TOA flux. Loeb et al. (2020) show that while the magnitudes of clear-sky fluxes associated with the two definitions can be quite large, differences between their anomalies are relatively small.

TOA radiation changes for different cloud types are evaluated using the CERES FluxbyCldTyp Ed4.1-daily and -monthly products (Sun et al. 2022). The FBCT product has been used previously to generate observation-based cloud radiative kernels to quantify the sensitivity in TOA radiation to perturbations in meteorological conditions (Scott et al. 2020; Oreopoulos et al. 2022; Wall et al. 2022; Myers et al. 2023), to study changes in cloud properties and radiative fluxes by cloud type as a function of convective aggregation (Xu et al. 2023), and to evaluate climate models (Eitzen et al. 2017). FBCT provides CERES Terra and Aqua daytime 1°-regional gridded daily and monthly averaged TOA radiative fluxes and MODIS-derived cloud properties (Minnis et al. 2008, 2011a, 2011b) stratified into 42 cloud types for 6 cloud optical depth and 7 cloud effective pressure intervals, as defined in Rossow and Schiffer (1991). The cloud types are defined from the vantage point of an observer in space that only sees the clouds that are exposed to space. Thus, cloud effective pressure is determined from the topmost portion of a cloudy column and optical depth corresponds to column optical depth (Cole et al. 2011). TOA fluxes are also provided for all-sky and clear-sky conditions. In FBCT, “clear-sky” corresponds to fractional area within a 1° × 1° region (gridbox hereafter) that is not covered by cloud. Since the FBCT uses Terra and Aqua, it only starts in July 2002 onwards. Accordingly, we consider 07/2002–12/2022 to assess changes in cloud fraction by cloud type.

### Changes in TOA Radiation by Cloud Class

To assess the influence of cloud changes on TOA fluxes, we develop a cloud classification scheme using 1° × 1° gridded daily mean estimated inversion strength (EIS) parameter (Wood and Bretherton 2006) provided in the SSF1deg Ed4.1-daily product (Doelling et al. 2013) and cloud type information from the FBCT Ed4.1-daily and -monthly products (Sun et al. 2022). EIS is derived from surface pressure, temperature and dew point temperature at 2 m, and temperature and geopotential height at 700 hPa provided in the GEOS-DAS V5.4.1 product (Rienecker et al. 2008).

We first produce a gridded monthly EIS-by-cloud-type dataset from the SSF1deg Ed4.1-daily and FBCT Ed4.1-daily products by sorting gridded daily EIS values into the 42 FBCT cloud types in each gridbox each day and averaging these monthly. The monthly EIS-by-cloud-type data are then used together with the FBCT-monthly product to determine cloud fraction and TOA flux gridbox averages for three low cloud type classes equatorward of 60° (Table 1). The three low cloud classes have cloud effective pressures > 680 hPa with EIS values > 5 K Stratucumulus (Sc), 0–5 K stratocumulus-to-cumulus transition (SCT), and < 0 K cumulus (Cu). This EIS stratification of low clouds is an estimate based upon the regional distribution of annual mean EIS, SW CRE, SST and vertical velocity at 700 hPa (e.g., see Fig. 1 from Myers and Norris 2015). In regions with EIS > 5 K, SW CRE is strongly negative, indicating that the clouds are bright, SSTs are cooler than surrounding regions, and subsidence is appreciable. These characteristics are consistent with stratocumulus (Wood 2012). Regions with EIS between 0 and 5 K exhibit weaker SW cloud radiative cooling, warmer SSTs, and weaker subsidence, consistent with stratocumulus-to-cumulus transition regimes. Low cloud areas with EIS < 0 K primarily occur in the tropical trade wind region over warm oceans where shallow cumulus typically reside. Middle and high cloud classes equatorward of 60° are defined for cloud effective pressures of 440–680 hPa and < 440 hPa, respectively. A polar cloud class is defined for all clouds poleward of 60°.

**Table 1:** Definition of cloud classes used to assess influence of cloud changes on ASR

Cloud class	Cloud top pressure (hPa)	EIS (K)	Latitude range
Stratocumulus (Sc)	> 680	> 5	60°S–60°N
Stratocumulus-to-cumulus transition (SCT)	> 680	0–5	60°S–60°N
Shallow cumulus (Cu)	> 680	< 0	60°S–60°N
Middle	440–680	–	60°S–60°N
High	< 440	–	60°S–60°N
Polar	–	–	90°S–60°S; 60°N–90°N

**Fig. 1:**
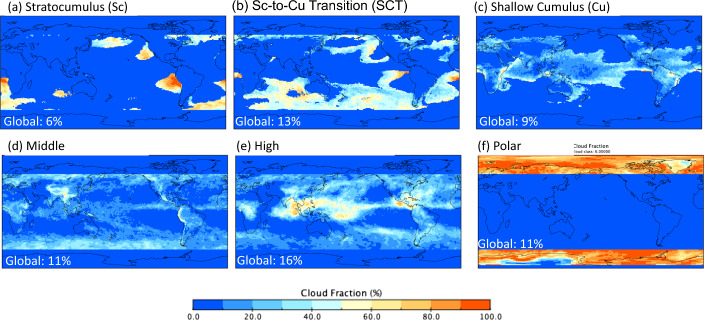
Cloud fraction by cloud class for September 2002. Global coverages of each cloud class are as indicated

The regional distribution of the cloud classes in Table 1 for September 2002 (Fig. 1a–f) shows that three low cloud classes exhibit a smooth transition from Sc off the west coasts of the Americas and southern Africa to SCT mainly over the Southern Oceans and Cu mainly over the tropics. Middle and high clouds are distributed throughout 60°S–60°N, but occur predominantly in the mid-latitudes and tropics, respectively. An important feature of this cloud classification scheme is that the cloud types that can occur in a gridbox vary from month-to-month. In contrast, Scott et al. (2020) assign only one cloud type per region for the entire period to define cloud regimes. Since clouds vary appreciably over short timescales (Oreopoulous et al. 2016), the identified cloud types should be allowed to vary in time to correctly represent TOA flux changes by cloud type.

Global statistics (Table 2) of each cloud category for a 20-year climatology (07/2002–06/2022) show that Sc has a large local area coverage (52%) and exhibits substantial variability, with a monthly SW TOA flux anomaly standard deviation of 4 Wm^−2^. However, the Sc cloud class accounts for only 7% of the globe, which reduces its global impact. Local cloud fractions for the low cloud types decrease from 52% (Sc) to 20% (Cu); while, SSTs increase from 281 K (Sc) to 300 K (Cu). These general characteristics are consistent with expectation for these cloud types (Wood 2012). Middle clouds have the smallest local fraction (13%) and weakest anomaly standard deviations compared to the other cloud types; while, polar clouds have largest local fraction and average SW flux, but the lowest OLR flux and SST.

**Table 2:** Local average and monthly anomaly standard deviation in coverage (fraction), SW and OLR TOA fluxes, and SST for clear-sky and the cloud classes in Table 1 for 07/2002–06/2022

	Local fraction (%)		SW TOA flux (Wm^−2^)		OLR TOA flux (Wm^−2^)		SST (K)		Global fraction (%)
	Avg	Stdev	Avg	Stdev	Avg	Stdev	Avg	Std	
Clear	34.1	0.47	53.7	0.36	271.1	0.47	290.0	0.16	34.0
Sc	52.2	1.45	113.9	4.16	242.1	2.09	281.1	0.74	7.0
SCT	40.9	0.71	95.2	1.67	257.1	1.22	289.6	0.40	12.7
Cu	20.2	0.46	97.0	1.06	276.8	0.79	299.6	0.27	8.9
Middle	12.5	0.22	117.1	0.85	234.5	0.68	293.1	0.16	11.1
High	20.4	0.38	125.2	0.97	202.3	1.09	293.1	0.16	18.2
Polar	76.6	1.17	157.6	1.76	198.9	1.31	266.0	0.43	8.1

A “local” average is determined from geodetic-weighted monthly averages of all 1° × 1° regions in which a given cloud type is observed. Also provided is the coverage of each clear or cloud class over the entire globe. Here, SSTs are from the CERES SSF1deg Ed4.1 daily product

### All-Sky TOA Flux Decomposition

The monthly mean all-sky TOA flux over a latitude range (*λ*_1_, *λ*_2_) and longitude range (*ϕ*_1_, *ϕ*_2_) can be expressed in terms of its clear and cloudy column contributions from 1° × 1° regions as follows:

1 $$\overline{F}_{{{\text{all}}}} = \overline{F}_{{{\text{clr}}}}^{{{\text{con}}}} + \mathop \sum \limits_{j = 1}^{n} \overline{F}_{j}^{{{\text{con}}}}$$

where ${\overline{F} }_{{\text{clr}}}^{{\text{con}}}$ is the monthly mean clear-sky column flux contribution and ${\overline{F} }_{j}^{{\text{con}}}$ is the monthly mean cloud column contribution for cloud class *j*, and n is the number of cloud classes. These are calculated as follows:

2 $$\overline{F}_{{{\text{clr}}}}^{{{\text{con}}}} = \frac{1}{W}\mathop \smallint \limits_{{\lambda_{1} }}^{{\lambda_{2} }} \mathop \smallint \limits_{{\phi_{1} }}^{{\phi_{2} }} \left( {1 - f_{T} \left( {\lambda ,\phi } \right)} \right)F_{{{\text{clr}}}} \left( {\lambda ,\phi } \right)w_{\lambda } d\lambda d\phi$$

3 $$\overline{F}_{j}^{con} = \frac{1}{W}\mathop \smallint \limits_{{\lambda_{1} }}^{{\lambda_{2} }} \mathop \smallint \limits_{{\phi_{1} }}^{{\phi_{2} }} f_{j} \left( {\lambda ,\phi } \right)F_{j} \left( {\lambda ,\phi } \right)w_{\lambda } d\lambda d\phi$$

where ${f}_{T}$ and ${F}_{{\text{clr}}}$ are the monthly gridbox total cloud fraction and mean clear-sky flux, respectively, and ${f}_{j}$ and ${F}_{j}$ are the monthly gridbox cloud fraction and mean flux for cloud class *j*. The total cloud fraction ${f}_{T}$ is equal to the sum of the individual ${f}_{j}$’s, and the weights ${w}_{\lambda }$ are geodetic weights whose sum $W$ over the domain is given by:

4 $$W = \int_{{\lambda_{1} }}^{{\lambda_{2} }} {\int_{{\phi_{1} }}^{{\phi_{2} }} {w_{\lambda } d\lambda d\phi } }$$

This decomposition of all-sky TOA flux represents all-sky TOA flux as the sum of area-weighted clear and cloudy column fluxes. Anomalies and trends in these contribution terms are impacted by area fraction and within column radiative property changes, but the sum is constrained to add to the corresponding all-sky value. We do not correct for non-cloud changes in the cloudy columns, nor do we attempt to remove ERF contributions. We expect that the cloud masking error is smaller than that for CRE since it is confined to the cloudy area only rather than a gridbox-wide difference between clear-sky and total-sky non-cloud contributions (Soden et al. 2008). We plan to extend the methodology to account for cloud masking contributions in the future.

### Validation of MODIS-Based Cloud Fraction Changes

To evaluate MODIS-based cloud fraction changes, Appendix 1 provides a detailed comparison of trends in MODIS cloud fraction by cloud type with those from coincident cloud-aerosol lidar and infrared pathfinder satellite observations (CALIPSO) cloud-aerosol lidar with orthogonal polarization (CALIOP) and CloudSat cloud profiling radar (CPR) data as provided in the CALIPSO-CloudSat-CERES-MODIS (CCCM) RelD1 product (Kato et al. 2010, 2011). The analysis in Appendix 1 shows that MODIS and CC cloud fraction trends are remarkably similar for each cloud type, providing confidence in the MODIS-based results. Additional comparisons between these and other cloud fraction products are provided in Stubenrauch et al. (2024, this collection), which focuses more on how well the different products agree in their regional cloud fraction distributions than on temporal variability.

## Results

### Global, Zonal and Regional Changes in TOA Radiation During CERES Period

As noted in Loeb et al. (2021a, 2022), the CERES record indicates that EEI has approximately doubled during the CERES period. During the first decade of CERES observations (03/2000–02/2010), EEI was 0.5 ± 0.2 Wm^−2^ and increased to 1.0 ± 0.2 Wm^−2^ for the most recent decade (01/2013–12/2022) considered here (Table 3). This is the result of a 0.9 ± 0.3 Wm^−2^ (≈0.4%) increase in ASR that is partially offset by a 0.4 ± 0.25 Wm^−2^ (≈0.2%) increase in outgoing longwave radiation (OLR). The corresponding change in incoming solar irradiance is negligible (0.02 ± 0.09 Wm^−2^). There is satellite evidence that the increase in EEI began during the decade prior to the CERES period based on a reconstruction of the earth radiation budget experiment (ERBE) record (Liu et al. 2020) and satellite altimetry and space gravimetry measurements (Marti et al. 2023).

**Table 3:** Average solar irradiance, ASR, − OLR and Net TOA radiation in Wm^−2^ for the first and most recent decades of CERES observations

	Solar irradiance	ASR	− OLR	NET
03/2000–02/2010	340.14	240.7	− 240.2	0.53
01/2013–12/2022	340.16	241.6	− 240.6	1.05
Difference	0.02	0.9	− 0.4	0.52

**Fig. 2:**
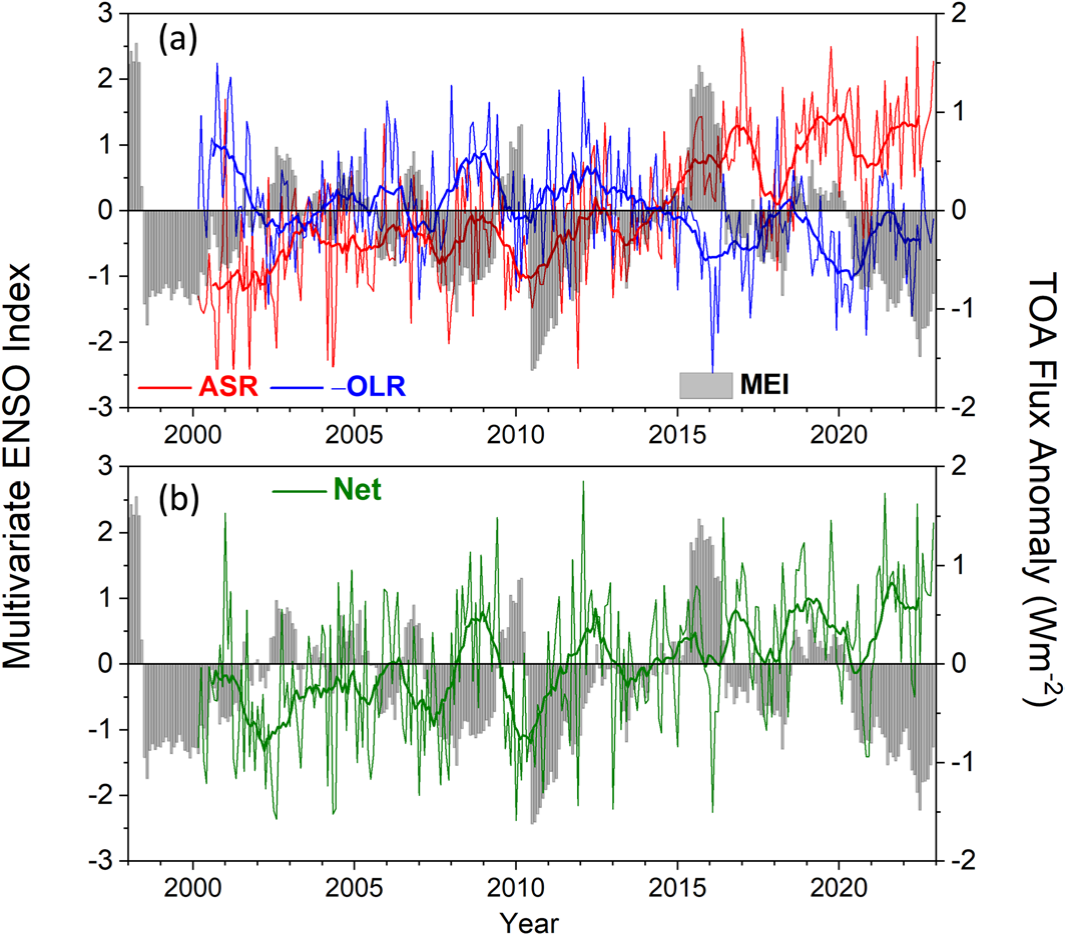
Global mean all-sky TOA flux anomalies and multivariate ENSO index (MEI) from CERES EBAF Ed4.2 for 03/2000–12/2022. **a** ASR and –OLR; **b** NET

Monthly anomalies in global mean TOA radiation show considerable variability superimposed over longer-term trends (Fig. 2a, b). Standard deviations in monthly anomalies for 03/2000–12/2022 are 0.7, 0.5 and 0.7 Wm^−2^ for ASR, –OLR and NET, respectively, and the corresponding trends are 0.71 ± 0.19, −0.26 ± 0.19, and 0.45 ± 0.18 Wm^−2^ per decade (uncertainties given as 2.5–97.5% confidence intervals). Monthly anomalies are consistent across CERES instruments on different platforms to < 0.2 Wm^−2^ (Loeb et al. 2018) and trends between Terra and Aqua, the two longest operating missions flying CERES instruments, agree to < 0.1 Wm^−2^ per decade (Loeb et al. 2022). Extensive validation of CERES instrument performance using a range of consistency tests involving different vicarious Earth targets and regular scans of the Moon provides further evidence that the CERES instruments are radiometrically stable (Shankar et al. 2023). The trends from CERES observations also agree with independently estimated trends from 0 to 2000 m ocean in situ data to < 0.1 Wm^−2^ per decade (Loeb et al. 2021a, 2022).

Analysis of atmospheric climate model simulations with a hierarchy of experiments using the GFDL CM4/AM4 suggest that the large positive ASR trend is due to additive contributions from ERF and climate feedback (radiative response) and the weaker negative trend in outgoing longwave radiation results from compensation between positive ERF and negative climate feedback contributions (Raghuraman et al. 2021; Hodnebrog et al. 2024). Since the ERF contributions add together and the climate feedback contributions offset one another, the model results suggest that ERF is the main driver of the positive trend in NET. However, the magnitudes of global TOA radiation trends in the climate model simulations are weaker than those in CERES, and there are large discrepancies in regional trend patterns. Furthermore, coupled climate models fail to represent observed SST patterns and associated feedbacks (Andrews et al. 2022; Kang et al. 2023; Olonscheck and Rugenstein 2024), adding to existing questions about the realism of climate model changes during the 21st Century (Trenberth and Fasullo 2009). These, together with substantial updates to SST and forcing datasets, provide additional motivation for further model–observation comparisons (Schmidt et al. 2023).

**Fig. 3:**
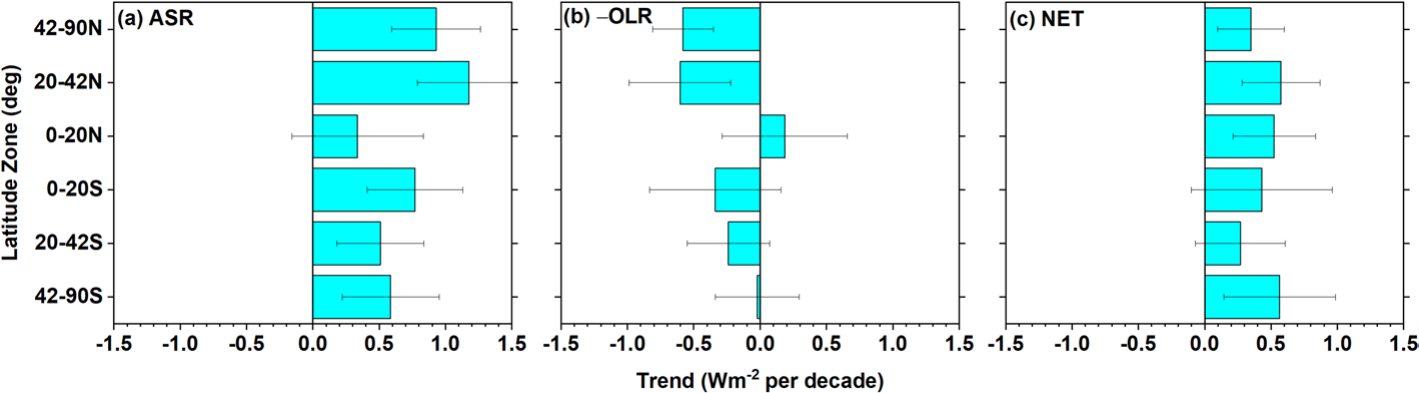
Zonal mean all-sky TOA flux trends for 03/2000–12/2022. **a** ASR; **b** –OLR; **c** NET

Zonal average trends for approximately equal-area latitude zones are positive for ASR and NET in the tropics, sub-tropics, and mid-high latitudes of both hemispheres; while, –OLR only shows appreciable negative trends in the NH sub-tropics and NH mid-high latitudes (Fig. 3a–c). Northern and southern hemisphere trends in NET are consistent to 0.06 Wm^−2^ per decade due to a compensation between weak ASR and –OLR hemispheric trend differences of opposite sign (Table 4). Datseris and Stevens (2021) also found hemispheric symmetry in reflected SW trends using CERES data for 03/2000–02/2020. Interestingly, GFDL AMIP climate model simulations fall within 0.2 Wm^−2^ per decade of CERES NH trends for ASR, –OLR and NET, but underestimate the ASR trend in the SH by −0.5 Wm^−2^ per decade due to erroneous trends in Antarctic sea ice and Southern Ocean cloud fraction, resulting in a much larger ASR hemispheric contrast (Raghuraman et al. 2021).

**Table 4:** Hemispheric and global trends in ASR, –OLR and NET for 03/2000–12/2022 in Wm^−2^ decade^−1^

	SH	NH	Globe	SH minus NH
ASR	0.62 ± 0.23	0.80 ± 0.22	0.71 ± 0.19	− 0.18 ± 0.36
− OLR	− 0.20 ± 0.21	− 0.33 ± 0.21	− 0.26 ± 0.19	0.13 ± 0.29
NET	0.42 ± 0.26	0.48 ± 0.21	0.45 ± 0.18	− 0.06 ± 0.31

Uncertainties are given as 2.5–97.5% confidence intervals

**Fig. 4:**
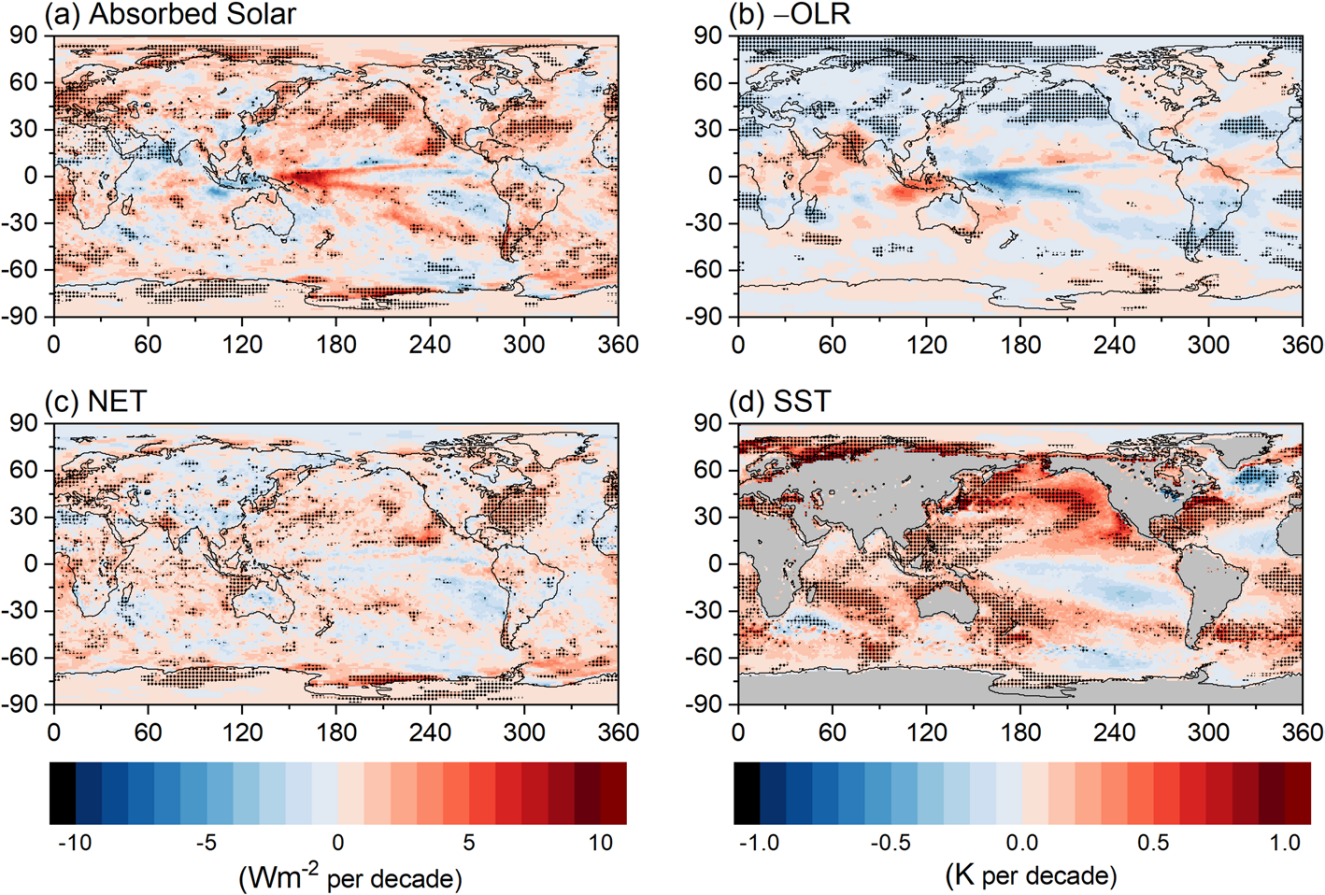
Regional trends in **a** ASR, **b** –OLR, **c** NET (Wm^−2^ per decade), and **d** SST (K per decade) for 03/2000–12/2022. Hatching indicates trends significant at 2.5–97.5% confidence level. SSTs are from ECMWF Reanalysis 5 (ERA5) (Hersbach et al. 2020)

Regionally, significant positive trends in CERES ASR occur off both coasts of North America, the Seas of Japan and Okhotsk, over the Arctic Ocean between the Kara and East Siberian Seas, the Southern Ocean to the east of South America, and Antarctica between 60° and 120°E (Fig. 4a). Large positive trends also occur over the equatorial Pacific Ocean, but because interannual variability is so large in this region due to the El Niño-Southern Oscillation (ENSO), the trends do not exceed the 2.5–97.5% confidence interval. Negative trends of –OLR, corresponding to increased thermal infrared emission to space, are appreciable over the NH eastern Pacific Ocean and over much of the Arctic (Fig. 4b). These regions are also associated with strong warming (Fig. 4d). Regional net radiation trends are positive over the NH Pacific, Indian and West Atlantic Oceans, but are mainly negative over the marine stratocumulus region off the west coast of South America (Fig. 4c). The similarity between the ASR and SST trend patterns is striking (Fig. 4a, d), particularly over the North Pacific, off the east coast of North America and west coast of South America.

**Fig. 5:**
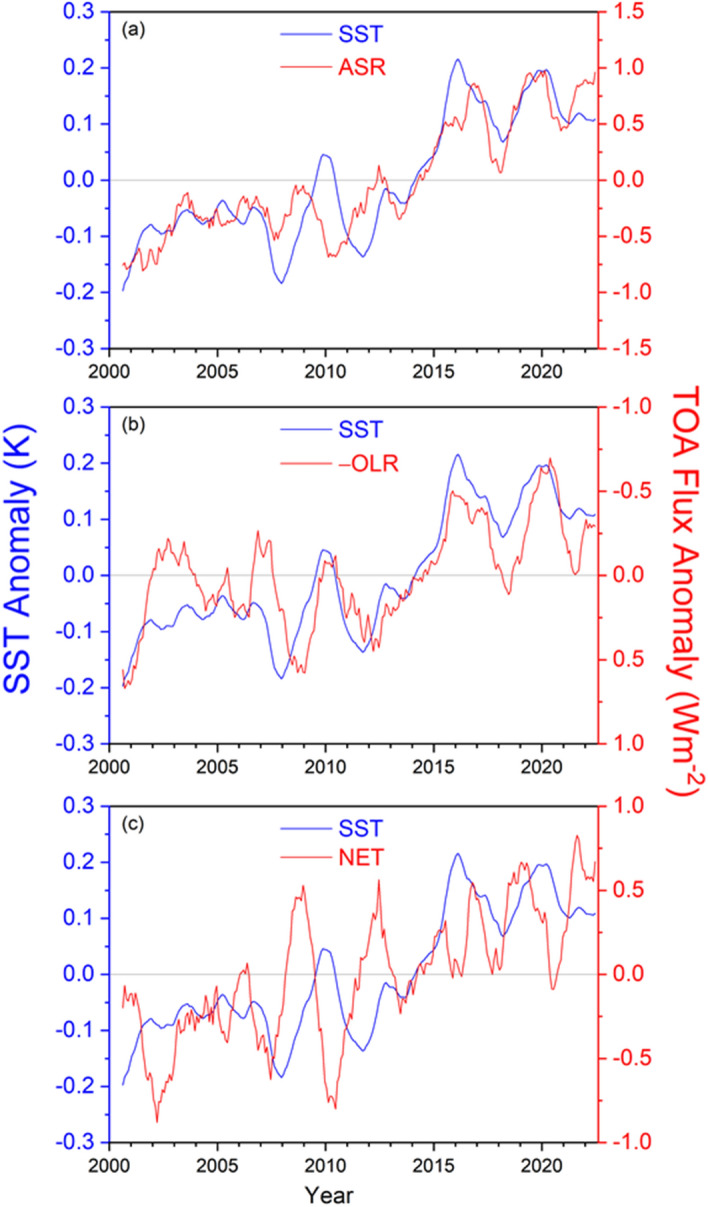
Twelve-month running average global anomalies in ERA5 SST and CERES **a** ASR, **b** OLR (positive up, since –OLR is displayed with a reversed y-axis), and **c** NET TOA radiation. Period considered: 03/2000–12/2022

Time series of global mean anomalies in SST, ASR, and –OLR also share similar features (Fig. 5a, b). In each case, twelve-month running average anomalies are relatively constant prior to 2010, and then increase sharply (decrease for –OLR) until a maximum is reached during the 2015–2016 El Niño event. The anomalies stay relatively flat after this event, albeit with considerable interannual variability. By comparison, the coherence at interannual timescales between anomalies in SST and NET radiation is much weaker (Fig. 5c) due to compensation between ASR and –OLR changes, but both do show a marked increase for the entire period.

Coupled climate models show a long-term trend in EEI and SST with anthropogenic forcing (Collins et al. 2013; Forster et al. 2021). Results in Fig. 5 confirm that increases in EEI and SST also occur in observations over a 20-year period despite substantial internal variability from heat exchange between the ocean mixed layer—which directly impacts SST—and the ocean layers below. Vertical ocean mixing has been shown to add considerable scatter between TOA radiation and SST trends at decadal timescales (Palmer et al. 2011).

### Changes During the Hiatus, Transition-to-El Niño, and Post-El Niño Sub-Periods

**Fig. 6:**
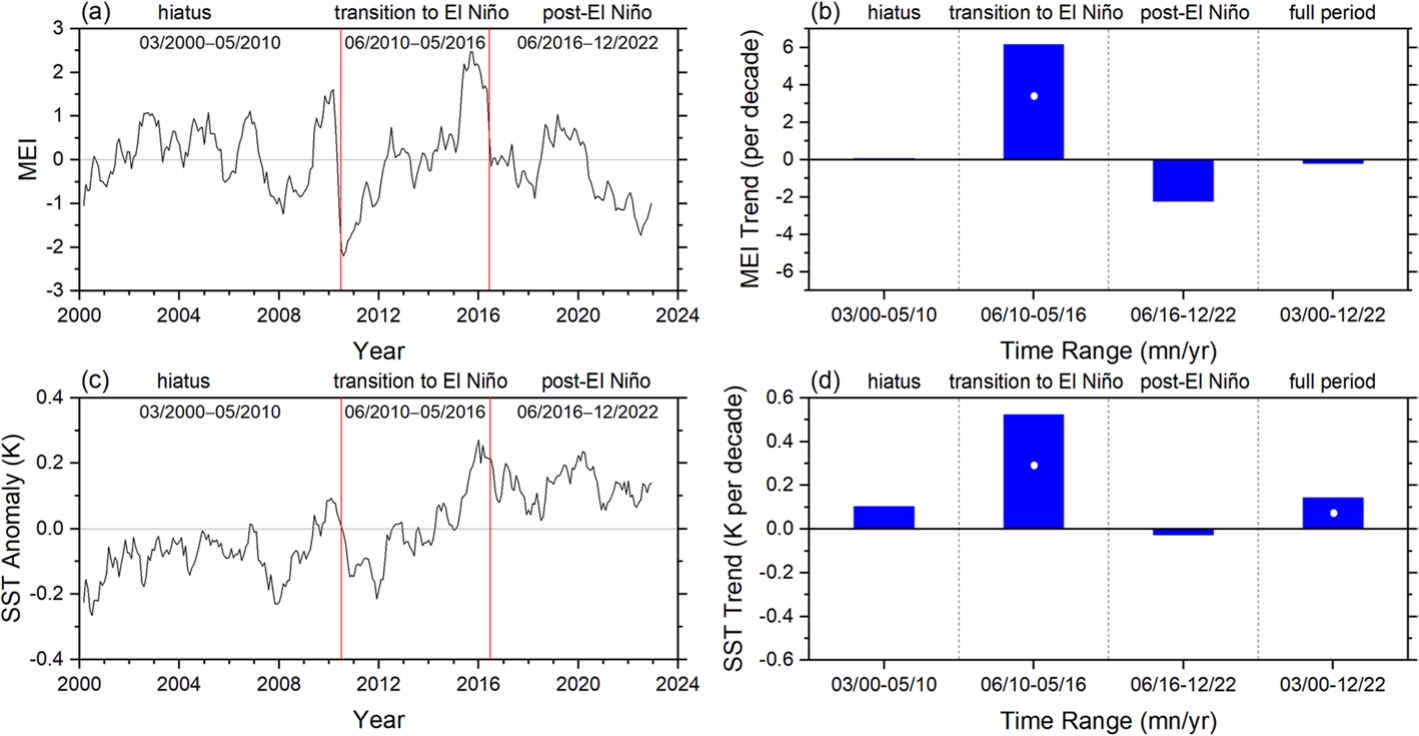
Monthly time series **a**, **c** and trends **b**, **d** for MEI (top) and anomalies in ERA5 SST (bottom). White circles in **b** and **d** correspond to trends that exceed the 2.5–97.5% CI. Time period 03/2000–12/2022

We examine the temporal evolution in SST and TOA radiation for the 3 sub-periods, which we define as follows: (i) “hiatus” (03/2000–05/2010), characterized by a negligible change in the Multivariate ENSO Index (MEI; Wolter and Timlin 1998) (Fig. 6a–d), a slower rate of global warming compared to the longer-term trend (Lewandowsky et al., 2015; Meehl et al. 2013; Trenberth 2015a) and to simulations from coupled climate models (Kosaka and Xie 2013); (ii) “transition-to-El Niño” (06/2010–05/2016), corresponding to the transition between the 2010–2012 La Niña and 2014–2016 El Niño events; and (iii) “post-El Niño” (06/2016–12/2022), corresponding to the transition between the 2014–2016 El Niño and the unusual extended 2020–2022 La Niña (so-called “triple-dip La Niña). During the “transition to El Niño” period, MEI and SST both show rapid increases that exceed the 2.5–97.5% CI (Fig. 6b, d). The SST trend during this period is 0.52 K decade^−1^, which exceeds the increase during the “hiatus” period by a factor of 5. For the entire period between 03/2000 and 12/2022, the SST trend is 0.14 ± 0.06 K decade^−1^ and the trend in MEI is near zero.

**Fig. 7:**
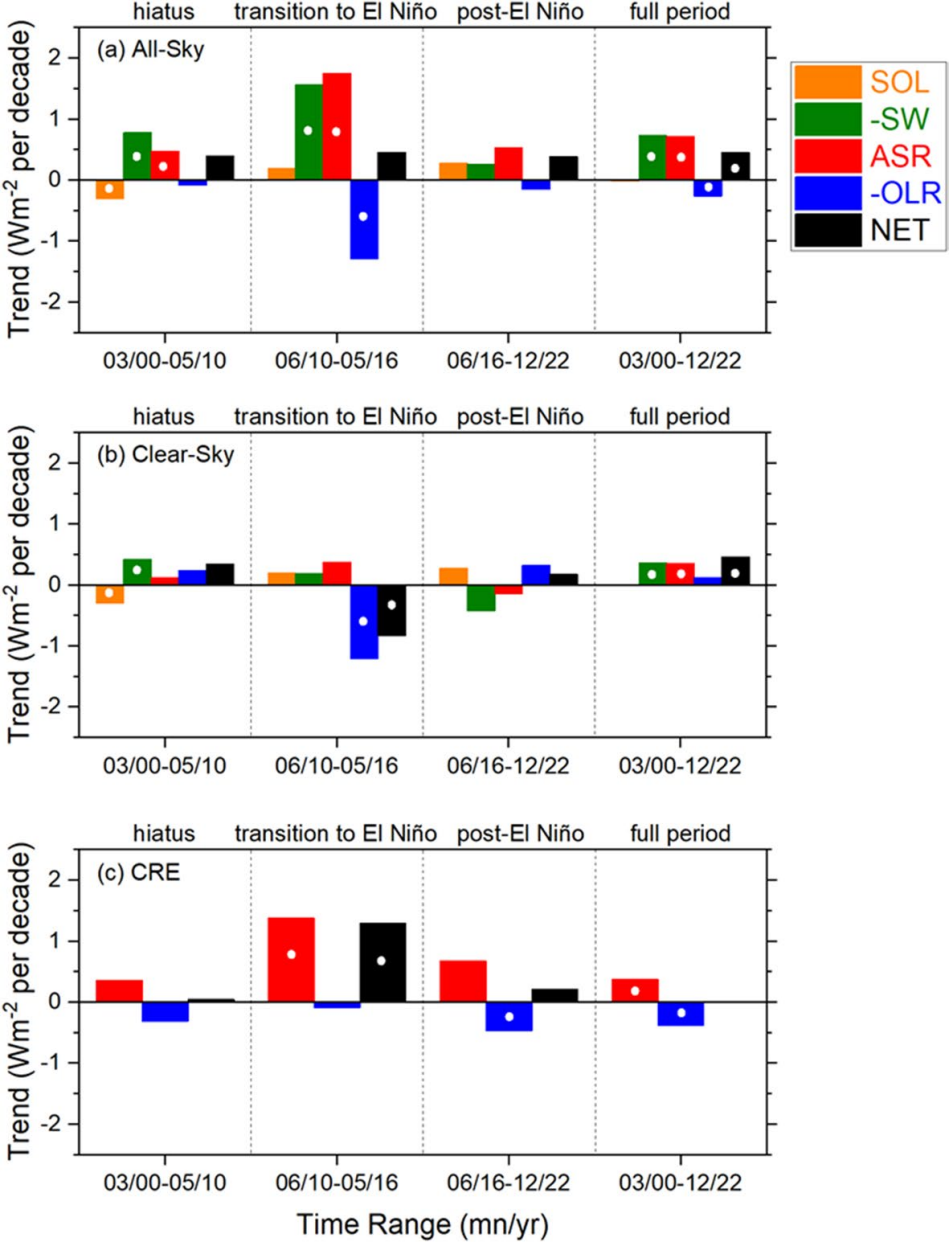
Trends in solar irradiance (SOL), –SW, ASR, –OLR, and NET TOA flux for **a** all-sky, **b** clear-sky and **c** CRE. White circles indicate trends that exceed the 2.5–97.5% CI

Trends in solar irradiance (SOL) and all-sky reflected SW (–SW, positive downwards), ASR, –OLR, and net radiation (NET) for the three sub-periods and entire time range (Fig. 7a) reveal that despite marked differences among sub-period trends for ASR, –SW and –OLR, reaching 1.3 Wm^−2^ per decade, NET trends remain within 0.1 Wm^−2^ per decade of one another and the trend over the entire period (0.45 Wm^−2^ per decade). During the “hiatus” the –OLR trend is near zero, so that the NET trend is determined by the difference between SOL and –SW. In contrast, all-sky –SW and –OLR both exceed 1 Wm^−2^ per decade in magnitude during the “transition-to-El Niño” period, but their sum (0.26 Wm^−2^ per decade) and the SOL contribution (0.19 Wm^−2^ per decade) add to ≈0.45 Wm^−2^ per decade for NET. This period is characterized by a substantial warming, leading to greater thermal emission to space from cloud-free areas (Fig. 7b). There is also a decrease in cloud fraction (not shown) that causes a strong ASR contribution by clouds (Fig. 7c), which compensates for the increased thermal emission. The trend in –OLR during the “post-El Niño” period is small, and SOL and –SW contribute approximately equally to the NET trend. In contrast to the all-sky case, clear-sky NET trends differ by up to −1 Wm^−2^ between sub-periods (Fig. 7b). Changes in clouds compensate for these differences under all-sky conditions, leading to a very similar all-sky NET trend in each sub-period.

It is unclear if the remarkable consistency among all-sky NET trends for the sub-periods occurs by chance or is a robust property of Earth’s energy budget. At shorter time scales than those defining these sub-periods, there is substantial interannual variability in NET radiation, as shown in Fig. 2b. Unfortunately, the CERES observational record is too short to test how robust these results are. Nevertheless, it implies a steady acceleration of climate warming since 2000.

It is noteworthy that NET CRE for the full period is near zero (Fig. 7c). Raghuraman et al. (2023) also show a negligible trend in what they describe as the “cloud feedback component of CRE”, which is obtained from the difference between CRE and the sum of ERF and cloud masking contributions. The implication is that net cloud feedback is not statistically significant during the CERES period. However, this conclusion assumes the model-derived ERF contribution to CRE is correct. The shortwave ERF contributions are primarily due to greenhouse gas adjustments and the aerosol-cloud indirect effects, both highly uncertain quantities (Smith et al. 2020). Furthermore, in Raghuraman et al. (2023) the model shortwave ERF contribution to CRE exceeds the longwave ERF contribution and accounts for as much as 57% of the total CERES SW CRE. In their observation-based PRP analysis Loeb et al. (2021a) found a significant positive trend in the cloud contribution to NET all-sky TOA flux, but aerosol-cloud indirect effects and greenhouse gas adjustments and were not removed from the cloud contribution. The uncertainty surrounding ERF thus makes it challenging to unambiguously isolate the net effect of clouds during the CERES period.

### TOA Radiation Changes by Cloud Type

The cloud classes (Sect. 2.2) and all-sky TOA flux decomposition (Sect. 2.3) provide a framework to assess TOA radiation changes by cloud type using FBCT. Since the CERES FBCT product uses data from both Terra and Aqua, the time period considered is limited to 07/2002–12/2022. Given that EBAF TOA global trends for this period are very similar to those for the full CERES period (Table 5), we expect results for the shorter period to be representative of the full period. We also find good agreement between EBAF and FBCT all-sky, clear-sky, and CRE trends for 07/2002–12/2022 (Table 5). The reason for the larger clear-sky –OLR difference is unknown. One contributing factor could be because of cloud mask differences as FBCT is a daytime-only product; while, EBAF uses both daytime and nighttime observations.

**Table 5:** Global trends in all-sky, clear-sky and CRE from EBAF and FluxbyCldTyp in Wm^−2^ decade^−1^

	03/2000–12/2022	07/2002–12/2022	
	EBAF all-sky	EBAF all-sky	FBCT all-sky
–SW	**0.73** ± **0.21**	**0.68** ± **0.25**	**0.67** ± **0.26**
–OLR	**–0.26** ± **0.19**	**–0.25** ± **0.22**	–0.20 ± 0.30
NET	**0.45** ± **0.18**	**0.47** ± **0.21**	**0.50** ± **0.23**

	EBAF clear-sky	EBAF clear-sky	FBCT clear-sky
–SW	**0.36** ± **0.11**	**0.32** ± **0.12**	**0.33** ± **0.12**
–OLR	0.12 ± 0.16	0.11 ± 0.19	0.29 ± 0.29
NET	**0.46** ± **0.14**	**0.46** ± **0.16**	**0.65** ± **0.19**

	EBAF CRE	EBAF CRE	FBCT CRE
–SW	**0.37** ± **0.18**	**0.36** ± **0.22**	**0.34** ± **0.20**
–OLR	**–0.38** ± **0.09**	**–0.36** ± **0.11**	**–0.49** ± **0.10**
NET	–0.008 ± 0.19	0.008 ± 0.21	–0.15 ± 0.20

Trends exceeding the 2.5-97.5 confidence interval are indicated in bold

**Fig. 8:**
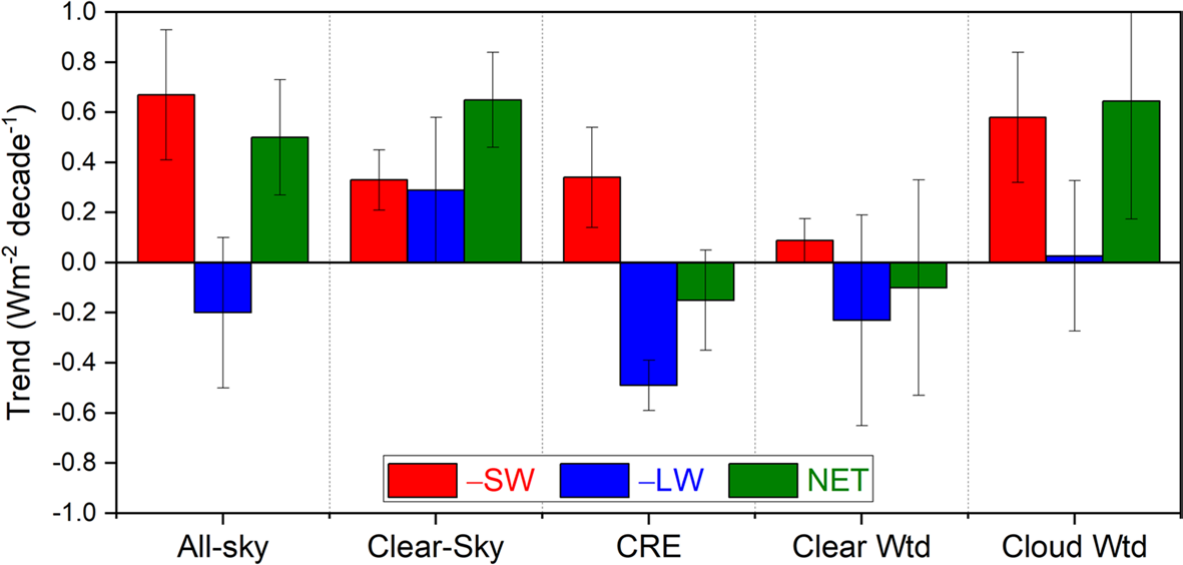
Trends in all-sky and clear-sky flux, CRE, clear fraction weighted clear-sky column (Clear Wtd) and cloud fraction weighted cloudy column (Cloud Wtd) flux contributions for –SW, –OLR (–LW), and NET TOA flux from FBCT product. Error bars correspond to 2.5–97.5% CI. Time period: 07/2002–12/2022

To illustrate the utility of the all-sky TOA flux decomposition framework, we compare global trends in TOA fluxes for all-sky, clear-sky and CRE alongside cloud fraction-weighted contributions computed using Eqs. (1–4) in Fig. 8. While the trend in net CRE is weak due to compensation between –SW and –OLR components, the trend for the area-weighted cloudy contribution is appreciable due to a large positive trend in –SW and negligible –OLR trend. Without any cloud masking adjustments in the cloudy regions, this result is already comparable to what is obtained using PRP analysis (see Fig. 2 in Loeb et al. 2021a). We expect that after subtracting cloud masking contributions, agreement with the PRP result will improve. After the corrections are made, trends in the –SW, –OLR and NET area-weighted cloudy contribution should decrease because part of the positive –SW trend is impacted by decreases in surface albedo from declining sea-ice coverage during the CERES period, and part of the –OLR trend is associated with reduced emission resulting from increases in water vapor and WMGG above the cloud top (Raghuraman et al. 2023). Results in Fig. 8 show that the all-sky decomposition approach in Sect. 2.3 provides a better framework than CRE for assessing the radiative impacts of cloud changes. The key difference with the CRE approach is that the all-sky decomposition separates changes from clear and cloudy areas whereas the CRE approach can only provide reliable results if there are no changes in cloud-free conditions, which is unrealistic.

**Fig. 9:**
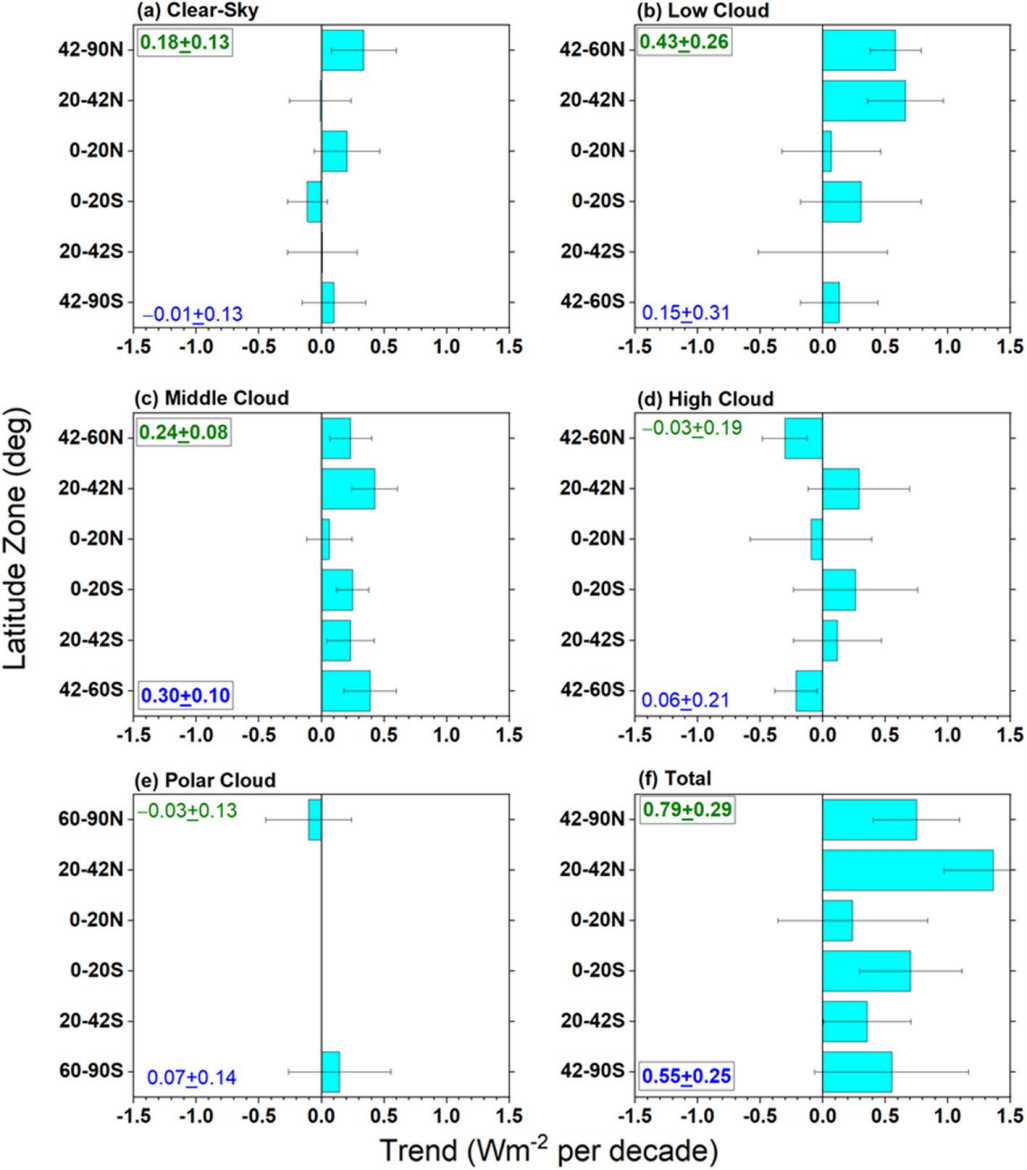
Contribution to zonal mean –SW trend from **a** clear-sky, **b** low cloud, **c** middle cloud, **d** high cloud, **e** polar cloud, **f** all. Period considered: 07/2002–12/2022. The SH and NH hemispheric average trends for each cloud type are indicated in each figure

**Fig. 10:**
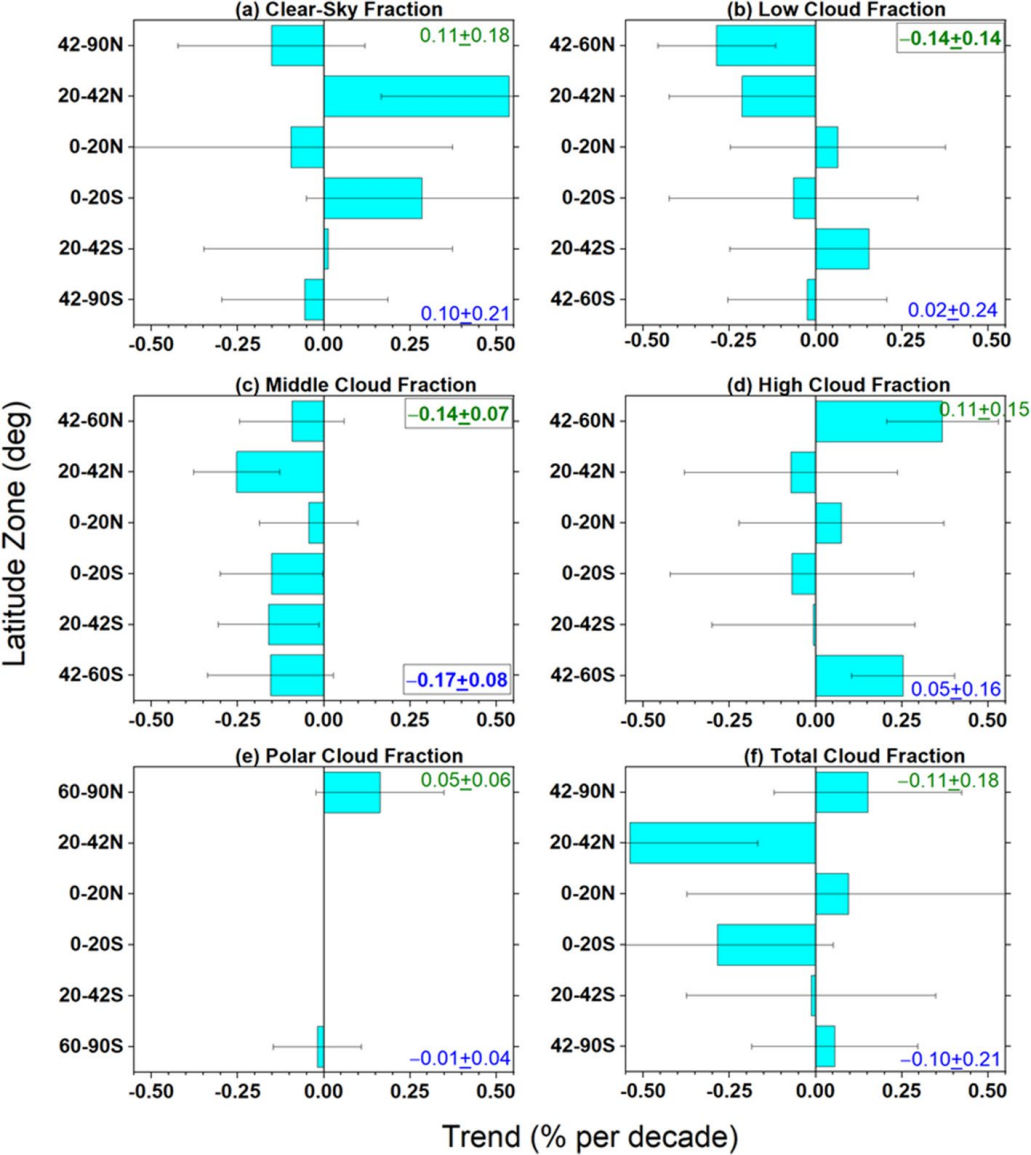
Same as Fig. 9 but for clear-sky and cloud fraction

**Fig. 11:**
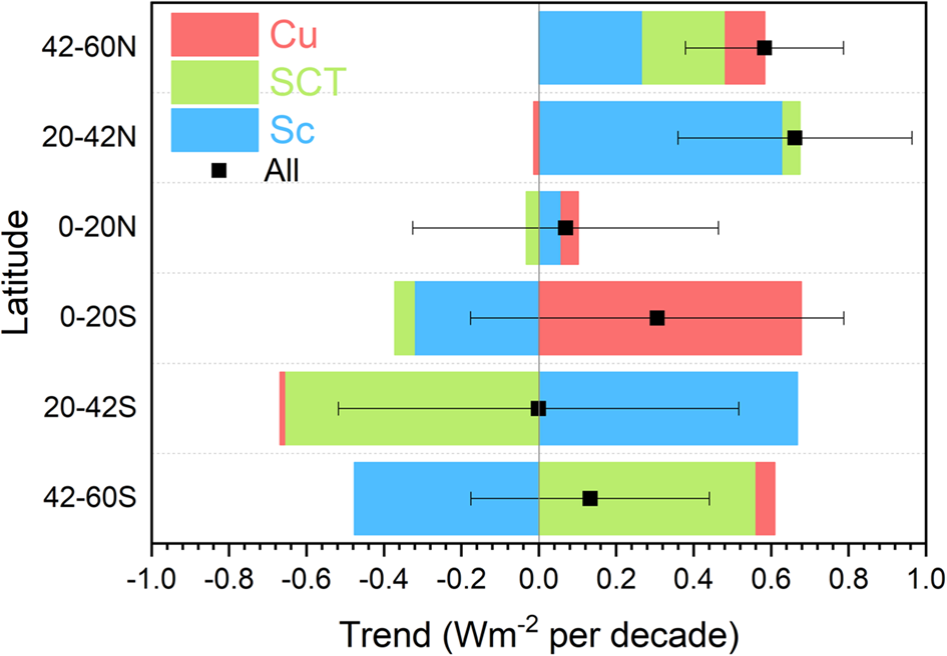
Zonal low-cloud trends with contribution from Cu, SCT and Sc. Period considered: 07/2002–12/2022

**Fig. 12:**
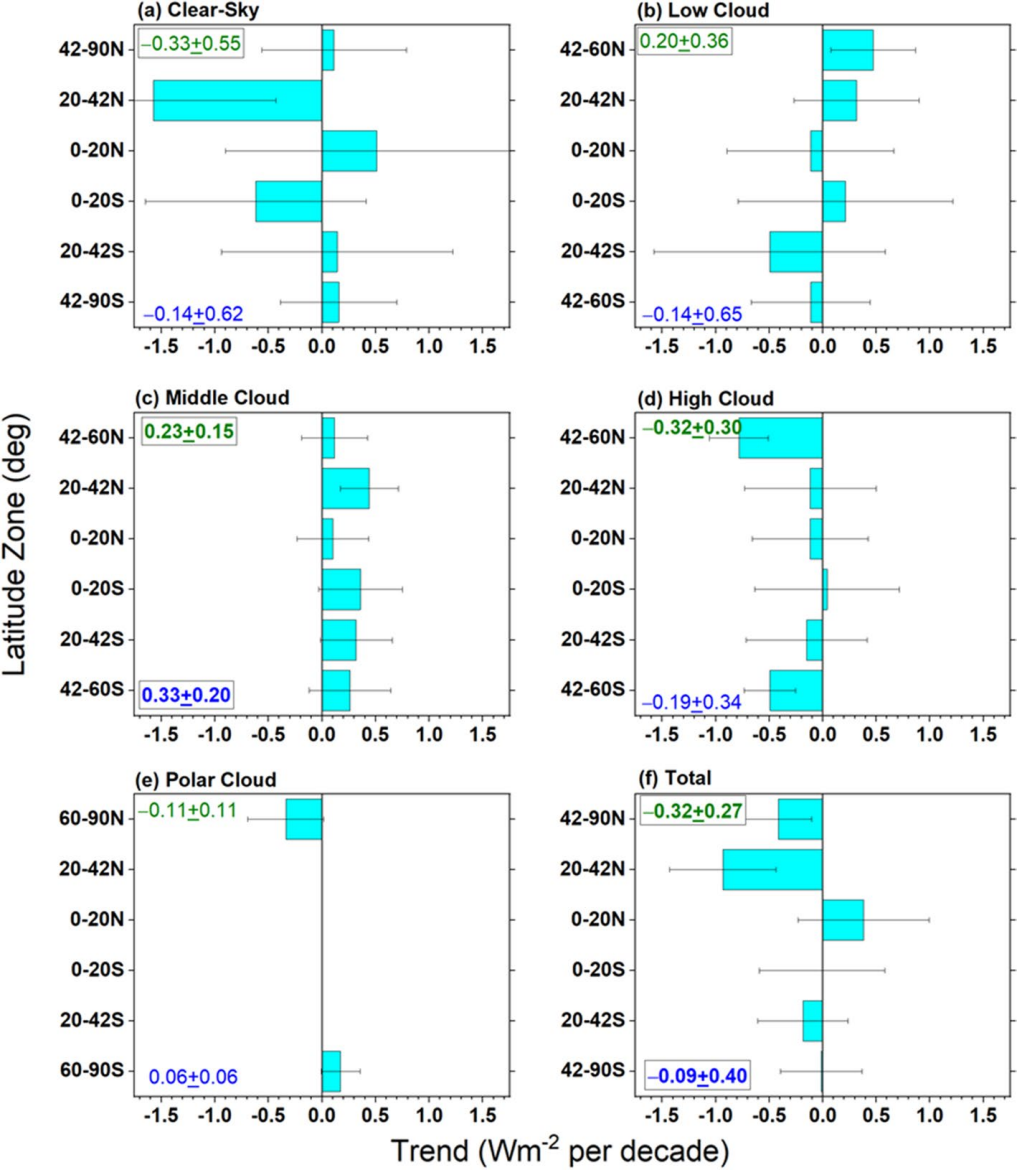
Same as Fig. 9 but for –OLR

TOA radiation and cloud fraction changes by cloud type for different latitude zones (Figs. 9, 10, 11, and 12) provide context for the hemispheric and global trends (Table 4). Since the contribution from each cloud class is an area fraction-weighted quantity over each latitude zone, the sum of all contributions plus the clear-sky contribution is equal to the total all-sky value. Decreases in low and middle cloud fraction and reflection between 20° and 60°N (Figs. 9b, c and 10b, c) and reduced reflection from cloud-free areas between 42° and 90°N (Fig. 9a) are the primary reasons for the NH ASR increase of 0.8 Wm^−2^ decade^−1^ in Table 4. Low cloud changes are primarily from Sc between 20° and 42°N; while Sc, SCT and Cu all contribute to the low cloud ASR increase between 42° and 60°N (Fig. 11). Regionally, these changes occur over the eastern and northern Pacific and off the east coast of North America, and coincide with large increases in SST (Fig. 4d). Other studies have noted the significant low-cloud response to SST in these regions (Myers et al. 2018; Andersen et al. 2023).

Interestingly, while there is a marked increase in clear-sky fraction in the NH sub-tropics between 20° and 42°N (Fig. 10a), the corresponding ASR trend contribution is near zero (Fig. 9a). This is likely because of a decrease in aerosol optical depth in this latitude range during the CERES period (Zhao et al. 2017; Paulot et al. 2018; Loeb et al. 2021b), which compensates for the increased clear-sky frequency, resulting in a near zero ASR trend contribution. While high clouds contribute little to the overall NH ASR trend, there is a notable increase in high cloud fraction between 42° and 60°N (Fig. 10d) that causes a negative ASR trend (Fig. 9d). Increased thermal emission in cloud-free conditions combined with high cloud changes contribute most to the –0.33 Wm^−2^ decade^−1^ NH –OLR change in Table 4. The increase in SST between 20° and 42°N likely contributes to a sharp increase in clear-sky thermal infrared emission (–OLR trend of –1.6 Wm^−2^ per decade) (Fig. 12a) while the increase in high cloud thermal emission between 42° and 60°N is associated with increased cloud fraction (Fig. 12d).

The ASR trend of 0.62 Wm^−2^ decade^−1^ in the SH (Table 4) is primarily associated with decreases in middle cloud reflection (Fig. 9c) and a weaker reduction in low-cloud reflection (Fig. 9b). Middle cloud fractions decrease by almost the same amount in each SH latitude zone (Fig. 10c); while, high cloud fraction increases between 42° and 60°S (Figs. 10d), resulting in a weak negative ASR trend contribution to ASR (Fig. 9d). In contrast to the NH, –OLR cloud trends in the SH are weak and largely cancel one another.

## Discussion

A key limitation of relying solely on observations to explain TOA radiation changes is that some of the underlying processes involved are difficult to isolate. For example, there is evidence that anthropogenic aerosol effective radiative forcing is weakening due to a decline in anthropogenic primary aerosol and aerosol precursor emissions (Quaas et al. 2022). Observations can provide estimates of the influence of aerosol-radiation interactions (Bellouin et al. 2005; Subba et al. 2020; Loeb et al. 2021b; Szopa et al. 2021), but the much stronger forcing contribution from aerosol-cloud interactions is more difficult to quantify as both clouds and aerosols are impacted by their environment (e.g., meteorology) in addition to having a two-way interaction between them (Gryspeerdt et al. 2016; McCoy et al. 2020). Furthermore, passive satellite aerosol retrievals are more uncertain in cloudy regions, and cloud retrievals are more uncertain in environments with abundant aerosol (Koren et al. 2007; Loeb and Schuster 2008; Gryspeerdt et al. 2016). This makes it challenging to unambiguously quantify how aerosol and cloud changes separately influence trends in ASR, which we show track closely with trends in SST, particularly over stratocumulus regions off the west coast of North America and over the North Pacific Ocean (see also Andersen et al. 2022; Myers et al. 2018). Establishing causality between observed SST and ASR changes also has its challenges as these share a two-way interaction (Trenberth et al. 2015b).

Nevertheless, progress is being made on the use of satellite observations for studying aerosols. A recent study by Wall et al. (2022) introduces a new method that removes confounding meteorological factors from observed sulfate–low-cloud relationships and narrows the uncertainty in aerosol forcing. Studies by Yuan et al. (2022) and Diamond (2023) use satellite observations to quantify the impact of sulfur regulations for shipping fuel on aerosol indirect forcing. Both studies find evidence for reduced radiative cooling by clouds following new regulations limiting sulfur emissions from the shipping industry by the International Maritime Organization 2020.

A longer TOA ERB observational record and new model output from CERESMIP provides new opportunities to determine how best to use observations and models for improving our understanding of the underlying process related to EEI changes. Current climate model simulations show similar patterns in regional TOA flux changes as observations, but the magnitudes of the changes differ markedly (Loeb et al. 2020), particularly over cloudy extratropical regions (Trenberth and Fasullo 2010; Zelinka et al. 2020). Similarly, the EEI trends from Raghuraman et al. (2021) are systematically lower compared to CERES. Conversely, if we find agreement between trends in TOA radiation in observations and climate model simulations, do they agree for the right reasons? To answer this, it will be necessary to use additional datasets and climate model output describing cloud and aerosol changes. Our comparisons with CC (Appendix 1) provide some confidence that the imager-based cloud changes are realistic. This means that there is some hope that meaningful comparisons between observed and model cloud changes is within reach.

## Summary and Conclusions

CERES observations show that Earth’s energy imbalance (EEI) has doubled from 0.5 ± 0.2 Wm^−2^ during the first 10 years of this century to 1.0 ± 0.2 Wm^−2^ during the past decade. This has led to accelerated increases in global mean temperature, sea level rise, ocean heating, and snow and sea ice melt. The increase in EEI is the result of a 0.9 ± 0.3 Wm^−2^ increase absorbed solar radiation (ASR) that is partially offset by a 0.4 ± 0.25 Wm^−2^ increase in outgoing longwave radiation (OLR). Since most of the energy added to the climate system associated with EEI ends up as heat storage in the ocean, changes in TOA radiation and ocean heat uptake (OHU) derived from in situ ocean data should track one another. Indeed, recently published analyses indicate that when in situ ocean measurements are supplemented with other data to fill in sparsely sampled regions, there is good agreement between variations and trends in OHU and CERES EEI for the Argo period between 2005 and 2019 (Loeb et al. 2021a; Hakuba et al. 2024, this collection).

Regional patterns of CERES ASR, –OLR and SST trends are similar, particularly over the North Pacific, off the east coast of North America and west coast of South America. Time series of global mean anomalies in SST, ASR, and –OLR also share similar features. In each case, twelve-month running average anomalies are relatively constant prior to 2010 (“hiatus” period), increase markedly (decrease for –OLR) prior to the 2015–2016 El Niño event (“transition-to-El Niño” period), and remain relatively flat after this event (“post-El Niño” period). Despite marked differences in global ASR and global –OLR trends between these sub-periods, NET trends remain strikingly within 0.1 Wm^−2^ per decade of one another. Since climate stabilization requires the climate forcing or net radiative imbalance to restore to zero, an increase in Earth’s radiative energy imbalance implies an acceleration of climate change rather than a continued, steady heating implied by a constant imbalance (e.g., von Shuckman et al. 2023). However, we note that NET radiation exhibits appreciable internal variability at interannual time scales. A longer observational record is needed to determine how robust these findings are.

We compare global trends in TOA fluxes of CRE alongside an alternate approach that uses the CERES FluxbyCldTyp (FBCT) product to isolate the cloudy and clear-sky contributions to all-sky TOA flux trends. While the trend in net CRE is weak due to compensation between –SW and –OLR components, the trend for the cloudy sky contribution is appreciable due to a large positive trend in –SW (i.e., reduced cloud reflection) and negligible –OLR trend. The latter is comparable to what is obtained using the PRP method and thus provides a better framework than CRE for assessing the radiative impacts of cloud changes. Further refinement would be required to account for cloud masking contributions in cloudy areas. Isolating the cloud contribution also requires removing the contribution from effective radiative forcing (aerosol-cloud indirect effects and greenhouse gas adjustments), which is highly uncertain.

When the cloudy sky contribution is stratified by cloud type, we find that decreases in low and middle cloud fraction and reflection and reduced reflection from cloud-free areas in mid-high latitudes are the primary reasons for increasing ASR trends in the NH. Low cloud changes are primarily from Sc between 20° and 42°N; while Sc, SCT and Cu all contribute to the low cloud ASR increase between 42° and 60°N. In the SH the increase in ASR is primarily from decreases in middle cloud reflection and a weaker reduction in low-cloud reflection. Increased thermal emission in cloud-free conditions combined with high cloud changes contribute most to the increase in OLR.

Climate model AMIP simulations suggest that the larger ASR increase observed during the CERES period is due to additive contributions from effective radiative forcing (ERF) and climate response to warming and it is spatial pattern; while, the weaker OLR change is associated with compensation between increasing ERF from continued emission of well-mixed greenhouse gases and increased infrared cooling to space relating to the radiative response to warming (Raghuraman et al. 2021; Hodnebrog et al. 2024). Model-based attribution of the CERES results are limited in number because the CMIP6 protocol ends in 2014. The new atmospheric model intercomparison project (AMIP) simulations proposed as part of CERESMIP (Schmidt et al. 2023) will provide updated model simulations through 2021 and will use input data sets, greatly expanding opportunities to assess model performance and attribution of the observed EEI trend.

## Figures and Tables

**Fig. 13:**
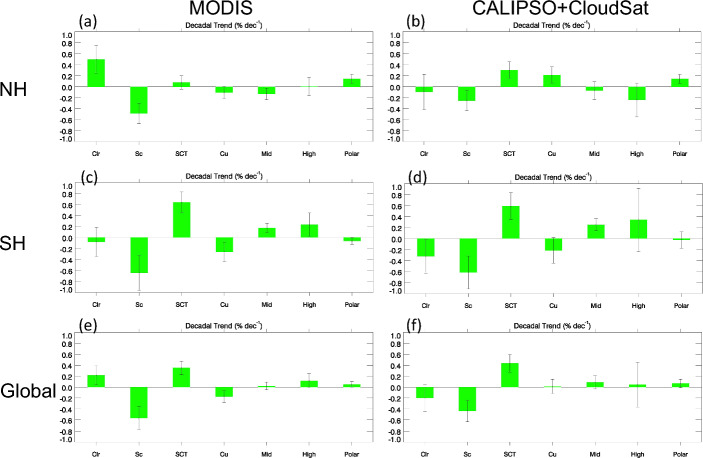
Clear-sky frequency and cloud fraction trends by cloud type from: **a** MODIS for NH, **b** CC for NH, **c** MODIS for SH, **d** CC for SH, **e** MODIS for globe, and **f** CC for globe using coincident measurements from 01/2008 to 12/2017

## Cloud fraction trend comparison between MODIS and CC

We compare MODIS-based cloud fraction trends with those from CALIPSO and CloudSat provided in the CCCM RelD1 product (Kato et al. 2010, 2011). The period considered is 01/2008–12/2017. As CALIPSO and/or CloudSat measurements are unavailable ≈20% of the time after 2011, we only include months in which all three instruments provide valid measurements. To ensure consistent spatial sampling, we only use MODIS cloud properties from CERES footprints that are collocated with the CALIPSO and cloudsat (CC) satellite tracks. MODIS cloud fraction is determined for each MODIS pixel using the CERES cloud algorithm (Minnis et al. 2021). The CALIPSO cloud mask is from CALIPSO vertical feature mask (VFM) version 4 product (Vaughan et al. 2009) with a threshold of the cloud-aerosol discrimination (CAD) score ≥ 20 and a horizontal averaging scale for cloud detection ≤ 20 km. Since CALIPSO detects optically thin ice clouds that are often missed by MODIS, we exclude optically thin ice clouds using the following criterion: if the cumulative cloud optical depth (*τ*) from the top is smaller than 0.3, the CALIPSO cloud layer is removed and treated as clear. For consistency, a *τ* filtering (*τ* ≥ 0.3) is also applied to MODIS. We find that the MODIS cloud trends with and without the *τ* filtering are nearly identical (not shown), meaning that the occurrence of *τ* < 0.3 is small. The CloudSat cloud mask is from the CloudSat 2B-GEORPOF release 5 (R05) product (Sassen and Wang 2008) with a threshold of the cloud mask value ≥ 30 and the radar reflectivity > -25 dBZ. The radar reflectivity condition is considered to minimize the impact of the degradation of the CloudSat cloud profiling radar (CPR) sensor (Mathew Lebsock, personal communication). To combine CC cloud layers we choose the closest CloudSat pixel for a given CALIPSO pixel.

After merging CALIPSO and CloudSat cloud layers, the cloud top height of the uppermost layer is used to assign the cloud type. This is because MODIS usually detects the uppermost cloud layers in the case of multi-layered clouds. The CC cloud top height is converted into the cloud top pressure using pressure profiles of the Global Modeling and Assimilation Office (GMAO)’s Goddard Earth Observing System Data Assimilation System (GEOS-DAS V5.4.1) product (Rienecker et al., 2008).

To evaluate MODIS cloud fraction trends, we compare coincident MODIS and CC during the common period from 01/2008 to 12/2017 for the same cloud types (Fig. ^13^a–d). Since this comparison is for a much shorter period, these results need not match those in Fig. 10. Furthermore, because −20% of the CC data after 2011 are missing, the trends may not even be representative of 2008–2018. Rather, the intent is to provide an independent assessment of the MODIS results using CC.

Cloud changes inferred from CC are sensitive to the cloud selection criteria applied in the analysis. For example, if we include CALIPSO clouds with small cloud optical depth values (< 0.3), high cloud trends become increasingly negative (not shown). In addition, the horizontal averaging scale for CALIPSO cloud detection also impacts the results. If CALIPSO water clouds with cloud top < 4 km are detected from a single lidar beam (1/3 km resolution) without horizontal averaging, decadal trends of low clouds are reduced relative to that where horizontal averaging is included. We estimate uncertainties in CC cloud fraction trends by combining three factors. The first factor is related to the uncertainty of the linear regression as standard errors (= *σ*_A_). The second factor is related to the uncertainty related to the *τ* filtering (= *σ*_B_). We estimate *σ*_B_ as the difference in the decadal trends with and without the *τ* filtering. The third factor is related to the uncertainty related to the horizontal averaging scales of CALIPSO water clouds below 4 km (= *σ*_C_). We estimate the value of *σ*_C_ as the difference in the decadal trends with 1/3 km scales of clouds and with 1/3, 1, 5, and 20 km averaging scales of water clouds below 4 km. The overall uncertainty is determined by summing the individual contributions in quadrature (= (*σ*_A_^2^ + *σ*_B_^2^ + *σ*_C_^2^)^1/2^). These are given as error bars in CC cloud trends.

MODIS and CC show remarkably consistent cloud fraction trends for each cloud type in the SH (Fig. 13c, d). Both show a large negative trend in Sc and a large positive trend in SCT, and weaker Cu, Mid, High and Polar cloud trends. The large error bar for CC high clouds is due to a greater sensitivity to our approach used to filter out thin clouds with optical depths < 0.3 that are below the MODIS detection threshold (Sect. 2.1). Differences are larger for the clear-sky fraction trend with MODIS showing no trend and CC showing a decrease in clear-sky fraction. With the exception of the Polar cloud case, the NH MODIS and CC cloud trends are generally weaker than those in the SH and show less agreement. Both show a significant decrease in Sc, but the magnitude of the decrease is larger for MODIS. There is a large discrepancy in clear-sky fraction, with MODIS showing an increase and CC showing little change. At the global scale, the main features that stand out are the Sc and SCT trends, which MODIS and CC capture. These comparisons suggest that MODIS is capable of capturing large changes in cloud fraction, but weaker trends are more uncertain.
